# Computational Study on Thermal Motion Sensors That Can Measure Acceleration and Rotation Simultaneously

**DOI:** 10.3390/s22186744

**Published:** 2022-09-07

**Authors:** Kamran Siddique, Yoshifumi Ogami

**Affiliations:** Department of Mechanical Engineering, College of Science and Engineering, Ritsumeikan University, 1-1-1 Noji-Higashi, Kusatsu 525-8577, Shiga, Japan

**Keywords:** fluid dynamics, unmanned aerial vehicles, microelectromechanical systems (mems), cross-axis sensitivity, frequency bandwidth

## Abstract

In this study, a new technique has been proposed by numerical simulations by which multiple physical quantities can be simultaneously measured. The sensor is a modification of existing physical sensors such as a thermal motion sensor. Simultaneous measurement of acceleration and rotation is presented herein. Cross-axis sensitivity is employed such that output sensitivities observed at two perpendicular axes, X and Y sensor data, are related to the input physical quantities. The physics involved in measurement is similar to that of a conventional thermal accelerometer, hence the governing equations predicting the sensor response are based on the conservation of mass, momentum, and energy, and are discretized by using a commercially available software FLUENT. A series of computational studies are conducted and using these studies a novel idea is proposed in which the maximum temperature values are obtained at various positions around a heating source and are correlated with the applied acceleration and rotational speed. A parametric study is also presented to find the optimum distance between the heater and sensors. The influence of changing gas medium on the temperature curves has also been examined and it has been concluded that CO_2_ generates the maximum performance due to its higher density and lower viscosity.

## 1. Introduction

Sensor technology and development has been an important breakthrough in industrial science and engineering. Over the years, research has been conducted for the design and manufacturing of efficient, handy, and cost-effective sensors. The development of micro and nanotechnology has further enabled the miniaturization of these sensors while maintaining performance. Sensor applications have increased a lot over the years and multiple sensors are incorporated into engineering devices. The manufacturing and repairing cost of such a large number of sensor-installment is expensive and time-consuming. For that reason, a new idea has been proposed here, which would allow the measurement of multiple physical quantities by employing a smaller number of sensors. This may provide a new cost-effective and time-saving approach.

Accelerometers have diverse applications in the automotive, consumer electronics, and biomedical industries. Conventional accelerometers convert accelerations into electrical signals using several mechanisms, including piezoresistivity, piezoelectricity, and capacitive type. However, the use of a solid-proof mass in such devices imposes mechanical limitations on the amount of shock and the sensing range they can undergo. In contrast, the convective nature of a thermal accelerometer with no moving parts can increase the sensing range of such a device.

**Figure 1:**
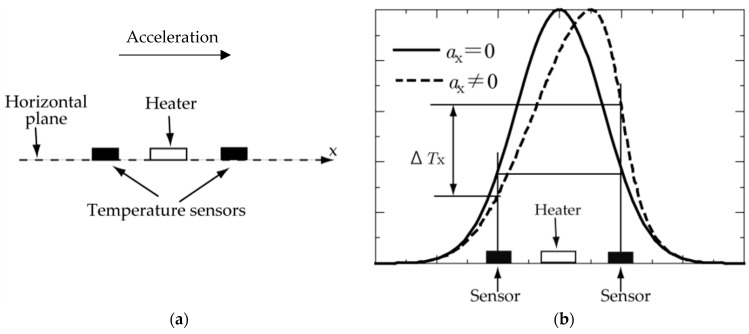
(**a**) Heater and temperature sensors; (**b**) temperature profile with and without acceleration (adapted from [1]).

**Figure 2:**
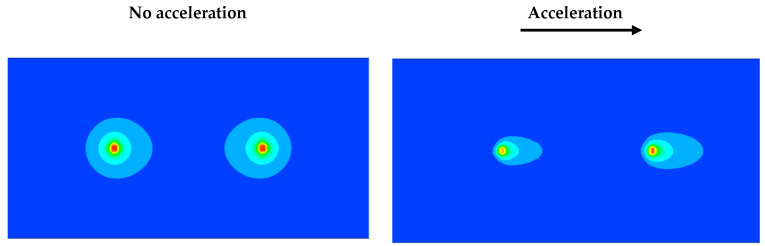
Temperature profile without acceleration (**left**) and with applied acceleration (**right**).

The concept of a thermal accelerometer is based on free-convection heat transfer. A heating source generates a consistent temperature profile that is altered by applied acceleration; thus, this difference in temperature Δ*T* is related to the change in acceleration, as shown in Figure 1. In this figure, the thermal sensor consists of a single heater source that heats the surrounding gas, creating a symmetrical temperature profile (solid line in (b)). When no acceleration is applied, the equally spaced sensors placed on the sides of the heater detect the same temperature. However, when acceleration is applied, the difference in temperature Δ*T* is modified, creating an asymmetrical temperature profile around the sensor (dashed line in (b)). Figure 2 depicts a clearer image of the concept in terms of the temperature contours and a side view of a cylindrical cavity with two heating sources. The red part denotes the highest temperature at the heating sources, and that around the boundaries (dark blue) is at room temperature (300 K). When no acceleration is applied, the thermal bubble is consistent around the heating sources. However, when acceleration is applied, the thermal bubble shifts in the direction of the applied acceleration. In this way, the pair of temperature sensors, placed equidistant from the heater, as shown in Figure 1, detect a difference in temperature, which is correlated with the change in acceleration.

One problem that has been pointed out recently is associated with the incorporation of multiple sensors into engineering devices. For micro- and insect-scaled unmanned aerial vehicles (UAVs), installing multiple sensors to measure each physical quantity not only imposes higher manufacturing and repair costs but can also be time-consuming. Therefore, to solve this problem, in our study, we propose the novel idea that in addition to acceleration, Δ*T* can also be correlated with other physical quantities, such as rotational speed, amplitude, and frequency of vibration. This technique does not involve creating a completely new device, but rather modifying existing motion sensors, such as thermal accelerometers. This is accomplished by considering the cross-axis sensitivity, which is the sensitivity observed in the plane perpendicular to the measuring direction relative to the measuring direction. Ogami [1] suggested that cross-axis sensitivity should not be removed but rather exploited. In this way, if multiple motion types are applied on a single axis, with sensitivities observed in other axes, the input physical quantities will have a relationship with the output sensitivities. In this study, a thermal motion sensor is considered; using cross-axis sensitivity, we can measure the acceleration and rotational speed simultaneously.

Because our thermal motion sensor involves physics similar to that of a regular thermal accelerometer, the performance parameters are also the same. The sensitivity and frequency bandwidth of any sensor device play important roles in defining its performance. Sensitivity is the quotient of the change in an indication of a measuring system and the corresponding change in a value of a quantity being measured. Frequency bandwidth is a measure of how quickly the sensor can respond to changes in input physical parameters. Over the years, various analytical studies and computational simulations have been conducted to predict the sensitivity and frequency bandwidth of microelectromechanical systems (MEMS) thermal accelerometers. The thermal accelerometer was first reported by Albert [2] in 1997. Goustouridis [3] developed a conductive thermal accelerometer comprising a polysilicon heater and two thermopiles. This device uses electrical energy as a parameter related to thermal energy. However, the literature has mentioned that obtaining the temperature profile using temperature sensors and relating it to the input thermal energy generates better results. Brahim [4] developed a 3D model for finite element method (FEM) simulations using a derived analytical model to study the conductive behavior of MEMS thermal accelerometers.

**Figure 3:**
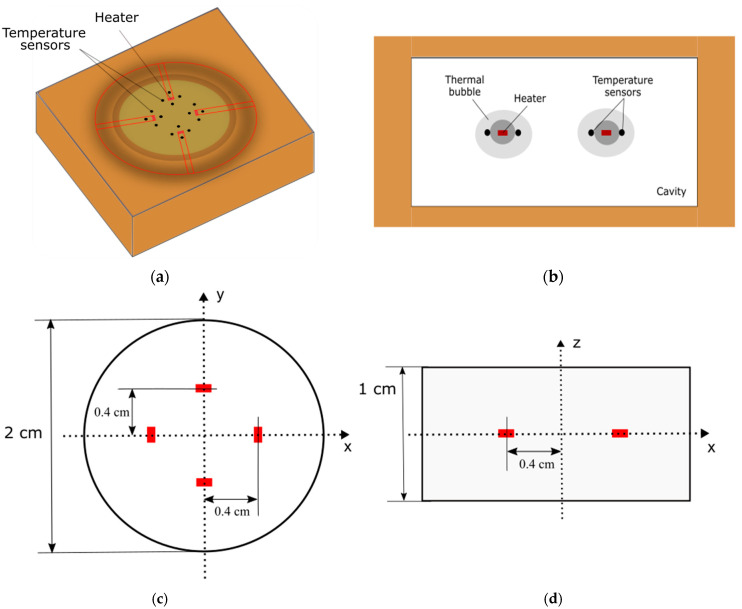
Model illustration: (**a**) isometric view; (**b**) side view and geometric dimensions: (**c**) cross-section view; (**d**) side view.

Researchers have developed theoretical, computational, and experimental models for improving the performance of thermal accelerometers. It has been observed that a high heating power and a large device size lead to an increase in sensitivity [5]. However, an increase in the pressure of the air medium results in a decrease in the frequency bandwidth. Additionally, gas media with high densities and low viscosities appear to result in better sensitivity [6]. Leung [2,7] demonstrated that the sensitivity of a thermal accelerometer is linearly proportional to the Grashof number (Gr):

(1) $$\mathrm{Gr}=\frac{{g\rho }^{2}{\beta L}^{3}\Delta T\;}{{µ}^{2}},$$

(2) $$\mathrm{Pr}=\frac{µ\;}{\alpha \;},$$

where g, ρ, β, L, ∆*T*, µ, and α are the applied acceleration, gas density, coefficient of volumetric expansion, characteristic size (generally denotes the cavity size; see Figure 3), temperature difference between the heater and the boundary of the sensor, kinematic viscosity, and thermal diffusivity, respectively. These parameters can be used to predict the device performance. From Equations (1) and (2), it can be observed that the sensitivity of the device can be significantly increased by using a high-density and low-viscosity fluid. The properties of some fluids and their calculated Gr and Prandtl (Pr) numbers are listed in Table 1 and Table 2, respectively. These parameters can be modified and optimized to increase the sensitivity of a thermal accelerometer.

As shown in Table 1 and Table 2, CO_2_ has the highest density and lowest kinematic viscosity; consequently, it has the highest Grashof number. In comparison, air has a lower density-to-kinematic viscosity ratio and hence a lower *Gr* value compared with CO_2_. High viscosity yields higher resistance to gas flow and, in return, lower sensitivity.

The size and power of our target model were determined as follows: in the literature, different scales of UAVs have been studied to achieve lighter weight and lower lifting and sensing power. Kevin [9] built an 80 mg, insect-scale, flapping-wind robot with a power consumption of 19 mW. Another study specifies the lifting and sensing power to be 100 mW for an insect-scale UAV with a mass of 100 mg [10]. Therefore, keeping this in view, our target model consists of a device with dimensions of 1 cm (height) and 2 cm (diameter) with a heating power of 70 mW for application in small-scale UAVs and robots.

The design of the computational study is illustrated in Figure 3. Four sets of heaters were considered, and two sets of sensors were placed equidistant from the heaters in *x* and *y* directions (therefore, four sets of sensors for each heater). The changes in the input physical quantities correlate with the altered temperature distribution. Because the fluid under study is governed by the conservation of mass, momentum, and energy, the commercially available software ANSYS FLUENT 18.2, which is a reliable and accurate fluid simulation software, was used for the analysis. After the temperature responses corresponding to the input physical quantities were obtained by computational simulation, the next step was to find an inverse function, and the results were plotted on a 3D surface using MATLAB, a programming, and numerical computing platform. This inverse function is installed in the computing unit of a real thermal motion sensor so that the sensor can calculate the values of the input physical quantities from the measured temperature values.

## 2. Materials and Methods

A computational simulation was performed to observe natural convection, and changes in the temperature profile as acceleration and rotational speed were simultaneously applied to the computational model. The governing equations for predicting the temperature profile of our thermal motion sensor are based on the principle of conservation of mass, momentum, and energy, which are as follows:

(3) $$\frac{\partial \rho }{\partial t}+\nabla \cdot \left(\rho u\right)=0$$

(4) $$\rho \left(\frac{\partial u}{\;\partial t}\right)+u\cdot \nabla u=-\nabla p\;+\nabla \cdot I+\;f$$

(5) $$\rho C_{p}\left(\frac{\partial T}{\partial t}+u\cdot \nabla T=k\nabla^{2}T\right)$$

where u is the flow velocity vector field, ∇ is the spatial divergence operator, p is the pressure, I is the total stress tensor, and f denotes the body forces acting on the fluid. The parameters *C_p_*, ρ, and k are the specific heat, density, and thermal conductivity, respectively, of the fluid in the cavity.

As shown in Figure 3, for our computational study, four temperature sensors (black dots) adjacent to the four edges of rectangular heaters (red rectangle) were considered.

The computational fluid dynamics (CFD) package FLUENT was employed for the analysis using the finite-difference method to discretize the governing Equations (3)–(5). A parametric study can be conducted because the software accommodates changing flow (initial and boundary) and geometrical conditions. As shown in Figure 2, the thermal bubble around the heater changes with changing acceleration. In this study, we observed that with the application of rotation, the thermal bubble around the heater also changed in the direction of rotatory motion.

**Figure 4:**
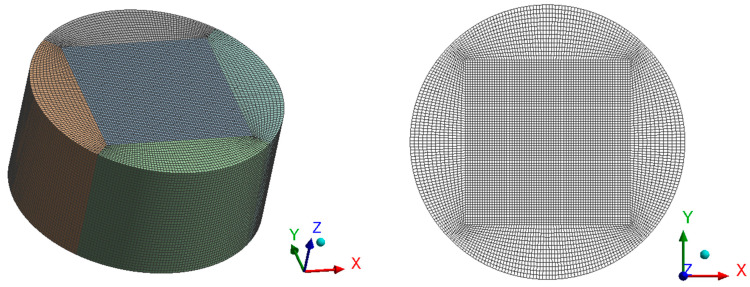
Computational mesh; isometric view (**left**) and wireframe top view (**right**).

An optimal grid design is required to obtain accurate results with a reduced computational time. To achieve this, we employed the grid resolution method proposed by Minhyung [11] and created a computational grid, as shown in Figure 4, using the meshing software ICEM in ANSYS 18.2. The number of elements and nodes of this structure are 310,947 and 1,283,732, respectively.

As seen in Figure 4, a simple cylindrical geometry for our device has been created with a height of 1 cm height and a diameter of 2 cm, as explained above. Heaters and temperature sensors are incorporated into this design using user-defined functions (UDFs). In the UDFs, the locations of heaters and sensors are defined and tracked using their cell IDs, which are unique for every cell, even when the geometry is moving. Instead of tracing the coordinates of heating sources, the cell IDs of cells containing the heating sources are traced to determine the centroid location of these coordinates. In addition, using the ‘DEFINE_SOURCE’ UDF, heat is applied to the cells where the heat sources exist. Furthermore, temperature values are extracted by looping over the entire cells and locating the cell IDs of sensors.

In FLUENT, a pressure-based transient solver was used with the energy model because the flow was not highly compressible with a lower Mach number. As explained above, a heating power of 70 mW was applied in response to the device’s intended applicability to small-scale UAVs that require low sensing and lifting powers. The DEFINE_CG_MOTION UDF was used to define linear and rotational motion alterations.

For the material of the computational domain surrounding the cavity, polyvinylidene fluoride (PVDF) and polyimide were chosen to be best suited because of their good thermal heat resistance with high values of specific heat capacity (*C_p_*) and low values of thermal conductivity (k), as listed in Table 3.

## 3. Results and Conditions

### 3.1. Measuring Maximum Temperature Difference Values

**Figure 5:**
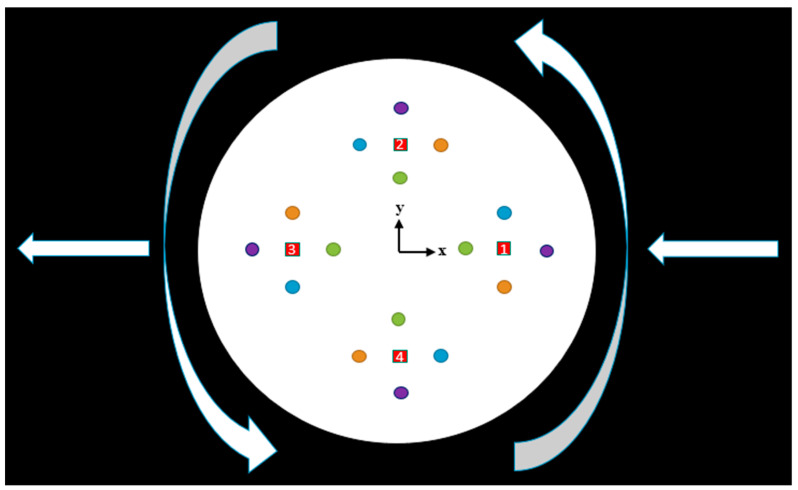
Direction of motion and position of heaters and sensors.

A computational study is conducted by obtaining temperature profiles with air as the gas medium when both rotations around the center of the cylindrical device in the counterclockwise direction and acceleration in the negative *x* direction are applied simultaneously, as shown in Figure 5. Four heating sources (red squares, numbered 1–4) were placed at various positions, and each heating source was surrounded by four temperature sensors (circles in four colors) in the *x* and *y* directions. As the device is accelerated and rotated simultaneously in space, each temperature sensor provides a unique temperature profile that changes with time. Sensors with the same color generate similar temperature responses. The sensor denoted *X21* is the *x* sensor around heater 2 in the positive direction (orange circle). Similarly, *Y42* denotes the *y* sensor around heater 4 in the negative direction (purple circle).

**Figure 6:**
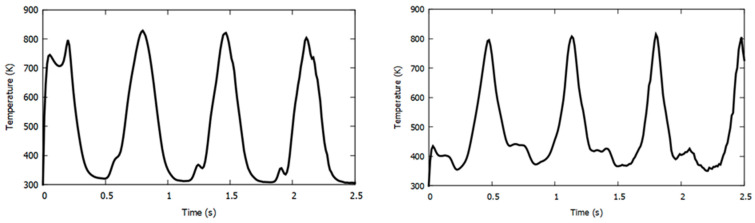
Temperature response of sensor Y31 (**left**) and sensor Y32 (**right**) at 3*a* and 3ω.

From the above figure, any set of one selected heater with its four surrounding sensors out of the four sets can be considered for the analysis. All sensors were placed equidistant from the heaters. The temperature response extracted from FLUENT for each sensor was observed to be periodic with simultaneous application of both acceleration and rotation. For an applied acceleration of 27.42 m/s^2^ (3*a*) and a rotational speed of 12.57 rad/s (3*π*), the temperature responses at sensors *Y31* and *Y32* around heater 3 (orange and blue) are shown in Figure 6.

To determine the relationship between the calculated temperature and both the applied acceleration and rotational speed, it is necessary to obtain the temperature difference Δ*T* (*T_Y32*–*T_Y31*) between the pair of sensors equidistant from the heater, which depends on both input quantities because of natural convection. Because the temperature response of all sensors is periodic, Δ*T* w. r. t. time will also be periodic, and we extract the maxima of that graph. For our analysis, we considered the results of the sensors around Heater 3. These temperature sensors are denoted *X31*, *X32*, *Y31*, and *Y32* (green, purple, orange, and blue in Figure 5), and the maxima of the temperature differences of the X and Y sensors are represented as Δ*T_X_max_* and Δ*T_Y_max_*, respectively. We studied the simultaneous application of accelerations from 9.81 m/s^2^ to 39.24 m/s^2^ (1*g*–4*g*) and rotational speeds from 6.28 rad/s to 15.71 rad/s (2π–5π).

**Figure 7:**
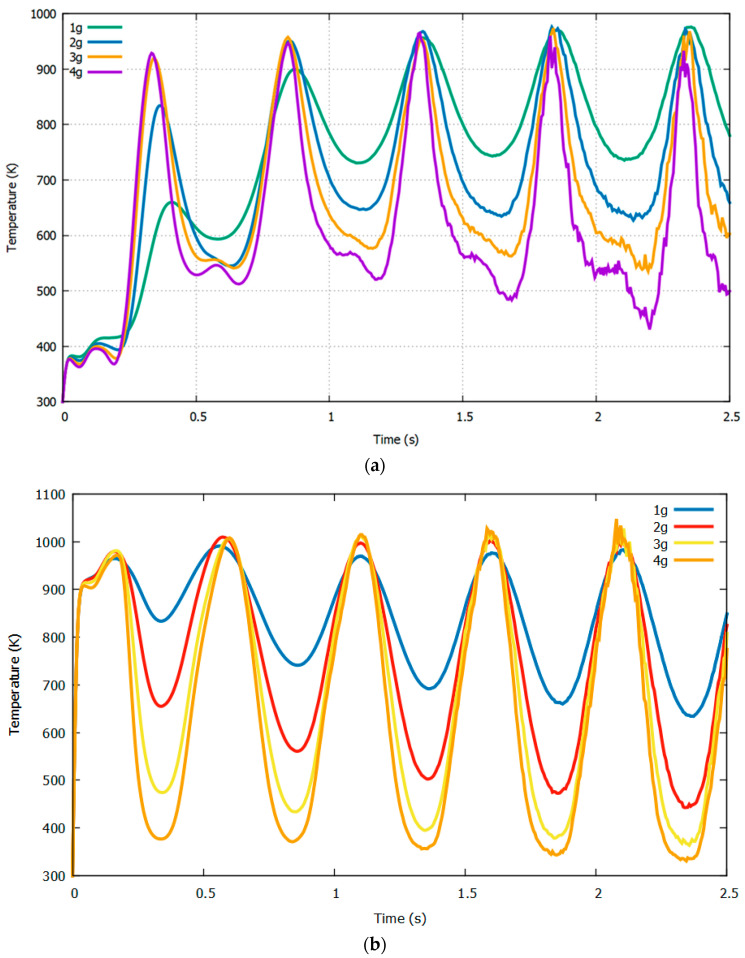
Temperature response of sensors *Y32* (**a**) and *Y31* (**b**) at 4*π* rad/s with varying acceleration.

**Figure 8:**
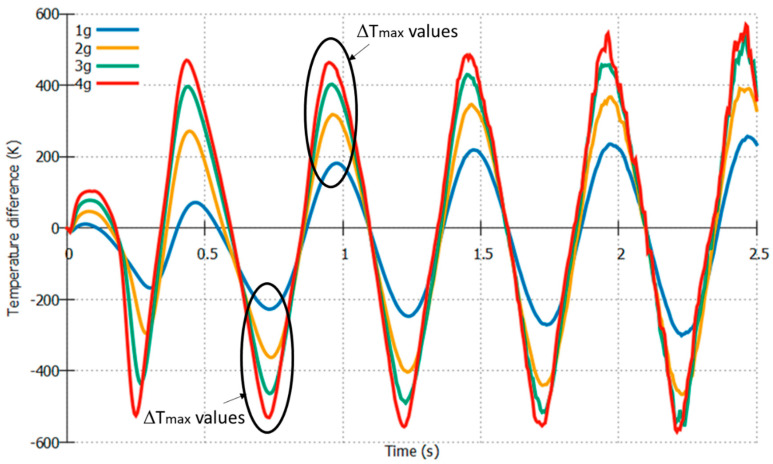
Temperature difference between sensors *Y32* and *Y31* w.r.t time at 4*π* rad/s with varying acceleration.

To visualize the above-mentioned method, we display the temperature responses at sensors *Y32* and *Y31* at a fixed rotational speed of 4*π* and varying accelerations of 1*g*, 2*g*, 3*g,* and 4*g* in Figure 7. The next step involved obtaining the difference between these two temperature responses, represented by *T_Y32*–*T_Y31*, as shown in Figure 8.

Furthermore, we extracted the maximum temperature values from Figure 8, which are denoted by Δ*T_Y_max_*. These maxima values change depending on the simulation conditions and parameters and directly influence the sensitivity of the device. Therefore, to improve the sensitivity, we studied the effects of changing the distance between the heater and sensors and changing the gas medium on the maximum temperature difference values (Δ*T_max_*). At 1*g*, 2*g*, 3*g*, and 4*g* with a constant rotation of 4π rad/s, the Δ*T_max_* values were 238.60852 K, 389.7796 K, 523.474 K, and 575.5737 K, respectively.

### 3.2. Study of the Effect of Changing Distance between Heater and Sensors

To investigate how changing the distance between the heater and sensors affects the maximum temperature difference (Δ*T_X_max_* and Δ*T_Y_max_*) and how the performance parameters can be improved, we considered six different positions of the temperature sensors by changing the distance from the heater to 0.0179, 0.0358, 0.0537, 0.0896, 0.1254, and 0.1433 cm. Based on these simulations, a suitable distance was determined.

**Figure 9:**
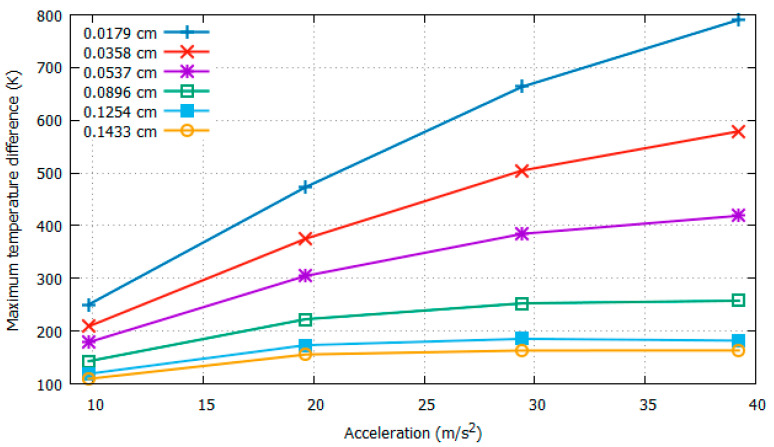
Δ*T_Y_max_* w.r.t acceleration at 12.57 rad/s with varying distance between heater and sensors.

**Figure 10:**
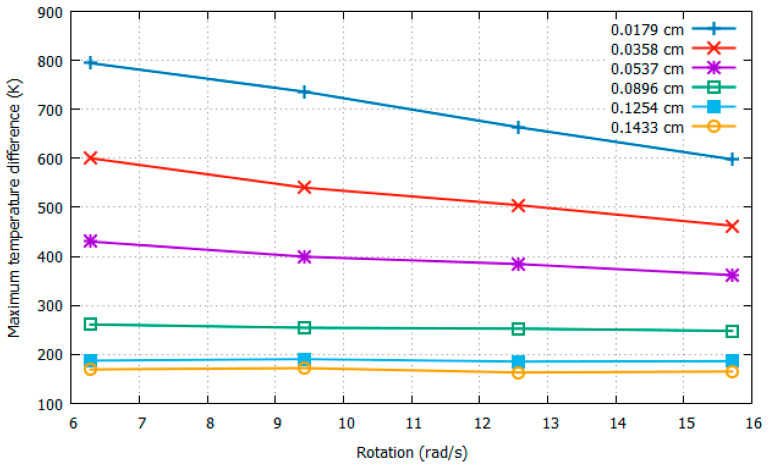
Δ*T_Y_max_* w.r.t rotation at 3*g* with varying distances between heater and sensors.

For the six different positions of the sensors, the Δ*T_Y_max_* values extracted at a constant rotational speed of 4π rad/s with increasing acceleration from 1*g* to 4*g* are shown in Figure 9, and those at a constant acceleration of 3*g* with increasing rotation speed from 6.28 rad/s to 15.71 rad/s are shown in Figure 10.

In Figure 9, with increasing acceleration, it can be observed that as the distance between the heater and sensor decreased, higher temperature values were detected. The point of interest is how these values are affected by changes in the acceleration. For the case of 0.0179 cm, when the acceleration was changed from 2*g* to 3*g*, Δ*T_Y_max_* increased from 473.0765 K to 663.5448 K, resulting in a difference in Δ*T_Y_max_* of 190.4683 K. However, for the case of a larger distance, i.e., 0.0896 cm, the temperature difference decreased to 30.0068 K. This means that with respect to acceleration, a shorter distance leads to better results (sensitivity).

In Figure 10, with increasing rotation, it is clear that, as the distance between the heater and sensors increased, the sensitivity decreased to a point where nearly straight lines in the graph are observed. In addition to sensitivity, resolution, which is defined as the smallest change in a quantity being measured that cause a perceptible change in the corresponding indication in measured quantity, is also clearly reduced by increasing the distance between the heater and sensors. Between 3π rad/s and 4π rad/s, the Δ*T_Y_max_* values for the case of 0.0358 cm were observed to be 540.3658 K and 504.6215 K, respectively, which shows a difference of 35.7443 K, as opposed to 4.9951 K for a longer distance of 0.1254 cm between the heater and sensors. Thus, a shorter distance between the heater and sensors generates better sensitivity and resolution with changing rotational speed.

**Figure 11:**
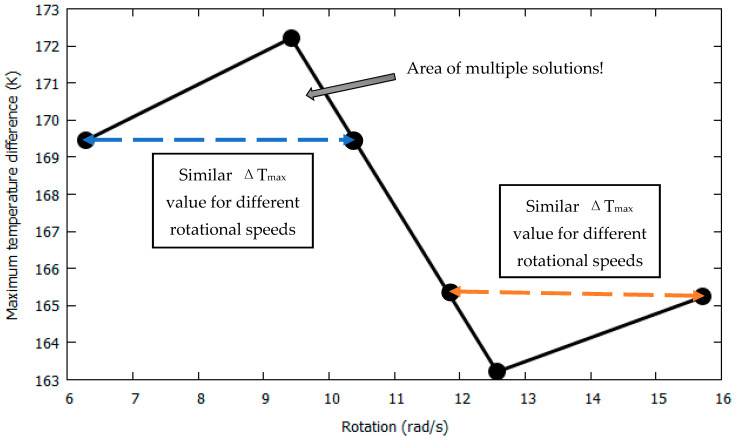
Δ*T_max_* w.r.t rotation at 3g with a distance of 0.1433 cm between heater and sensors.

However, it must be noted that the problem of multiple solutions is often encountered, as follows: for the case of the largest distance of 0.1433 cm, it can be observed in Figure 10 that the Δ*T_Y_max_* value decreased from 172.211 K to 163.2136 K with an increase in rotation from 9.42 rad/s to 12.57 rad/s. However, between rotations of 4π rad/s and 15.71 rad/s, it gradually increased from 163.2136 K to 165.2326 K. This means that the same Δ*T_max_* value exists for two different rotational speed values. This is illustrated in Figure 11. At rotational speeds of 6.28 rad/s and 10.41 rad/s, the Δ*T_max_* value is 169.451 K, and areas of multiple solutions can be observed. This is an undesirable problem, and to avoid it, it is necessary to obtain sets of data that provide unique solutions corresponding to unique input parameters.

It can be concluded from Figure 9 and Figure 10 that as the distance between the heater and sensor decreases, the sensitivities and resolution observed with respect to both acceleration and rotation increase. In addition, we observed the *uniqueness* of the solution for all six distances with changing acceleration. Conversely, with changing rotational speed, we observed *uniqueness* with distances from 0.0179 to 0.0896 cm between the heater and the sensors.

### 3.3. Study of the Effect of Changing Gas Medium

In addition to the distance between the heater and sensors, another important parameter that determines the quality of the result is the choice of gas medium. As discussed above, the sensitivity of a thermal motion sensor is dependent on the density and viscosity of the gas medium. The proportionality is shown in the following equation:

(6) $$\mathrm{Sensitivity}\;\propto \frac{\mathrm{Density}}{\mathrm{Viscosity}}$$

**Figure 12:**
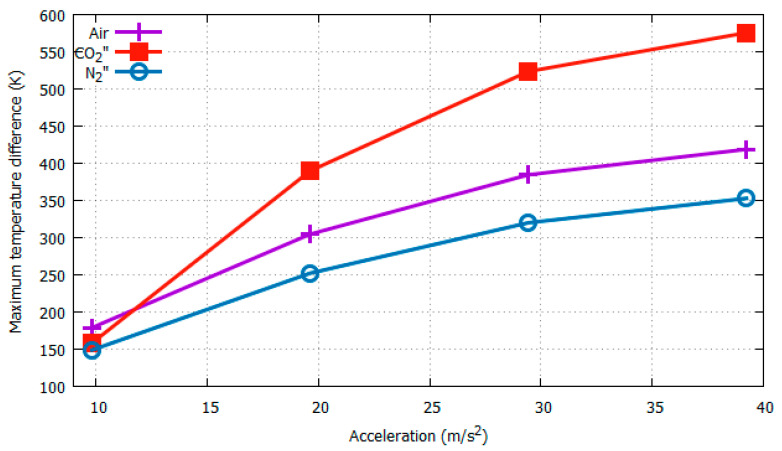
Δ*T_Y_max_* w.r.t acceleration at 12.57 rad/s with varying gas media.

**Figure 13:**
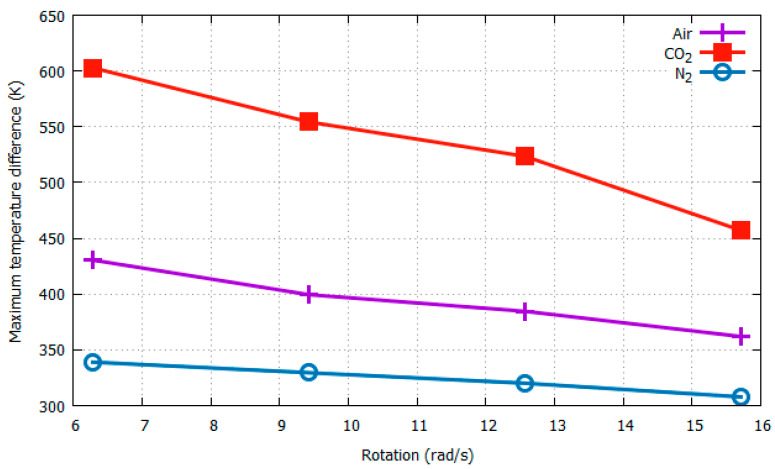
Δ*T_Y_max_* w.r.t rotation at 3*g* with varying gas media.

To study the effects of changing the gas medium, we considered three different gas media: air, N_2,_ and CO_2_. Their physical properties are listed in Table 1. For the computational simulations, we considered heater 3 with its four surrounding temperature sensors (Figure 6). The distance between the heater and sensors was set as the median value of the preceding simulations (0.0537 cm). The simulation results for Δ*T_Y_max_* with respect to acceleration at 3*g* and rotation at 4π rad/s are plotted in Figure 12 and Figure 13, respectively.

It can be seen from Figure 12 and Figure 13 that, as expected, the gas medium CO_2_ generates maximum sensitivity and resolution with both changing acceleration and rotation. In Figure 12, as the acceleration increased from 3*g* to 4*g*, the Δ*T_Y_max_* value jumped from 523.474 K to 575.5737 K, resulting in a difference of 52.0997 K, as opposed to that for N_2_, which had a difference of 32.8548 K. A more significant effect of the gas medium was observed when the rotation speed was changed from 12.57 rad/s to 15.71 rad/s, as shown in Figure 13. That is, CO_2_ generated a much larger difference in Δ*T_Y_max_* of 66.346437 K compared with that of 12.2239 K for N_2_.

**Figure 14:**
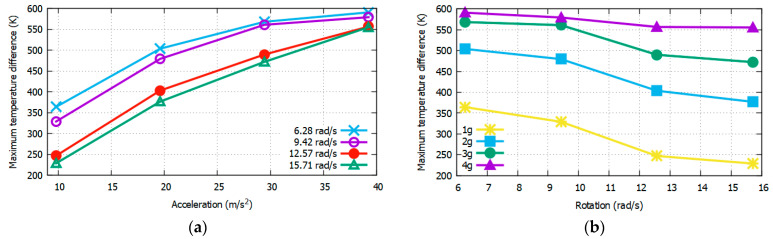
Δ*T_max_* at *X* sensors w.r.t acceleration (**a**) and rotation (**b**) at varying rotation values by using CO_2_ as the gas medium.

**Figure 15:**
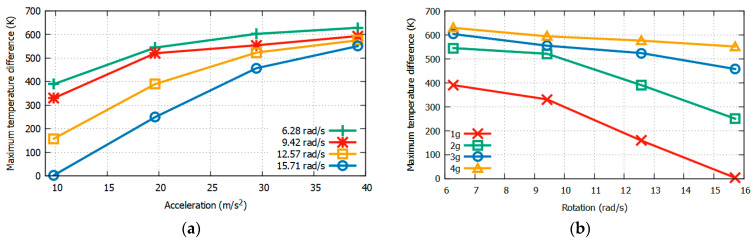
Δ*T_max_* at *Y* sensors w.r.t acceleration (**a**) and rotation (**b**) at varying rotation values by using CO_2_ as the gas medium.

Because the results in Figure 12 and Figure 13 were obtained only at 4π rad/s and 3*g*, respectively, to observe the consistency and reliability of the results under varying conditions, it is necessary to also obtain these maximum temperature values at wider ranges of rotational speeds and accelerations. The Δ*T_X_max_* and Δ*T_Y_max_* values around Heater 3 with respect to the acceleration (from 1*g* to 4*g*) and rotation (from 2π to 5π) using CO_2_ as the gas medium are listed in Table 4 and Table 5, respectively. The X- and Y-sensor results with changing acceleration and rotational speed from Table 4 and Table 5 are shown in Figure 14 and Figure 15, respectively.

It can be observed from Figure 14 and Figure 15 that the sensitivity is higher at lower rotation speeds and higher accelerations. Conversely, the resolution is higher at higher rotation speeds and lower accelerations.

### 3.4. Measuring Acceleration and Rotation from Maximum Temperature Difference Values

**Figure 16:**
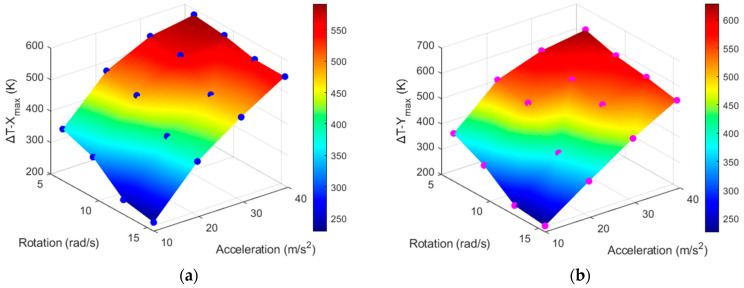
Δ*T_X_max_* (**a**) and Δ*T_Y_max_* (**b**) produced with the given acceleration and rotation.

Table 4 and Table 5 show that the values of Δ*T_X_max_* and Δ*T_Y_max_* are both related to the acceleration and rotation, and they can be measured by a real thermal motion sensor. Figure 16 shows three-dimensional plots of the values of Δ*T*_*X*_max_ and Δ*T_Y_max_* produced by acceleration (from 1*g* to 4*g*) and rotation (from 2π to 5π). The dots on both curves represent the data obtained by computational simulations, as shown in Table 4 and Table 5. Even though the acceleration was applied in the *x*-direction and rotation was around the *z* axis, because of cross-axis sensitivity, we observed temperature responses around both the X and Y sensors. This is expressed by the following relationship:

(7) $$(\Delta T_{x},\;\Delta T_{y})=f\;(a,\;\omega ),$$

where ω is the rotational speed in rad/s and *a* is the acceleration.

**Figure 17:**
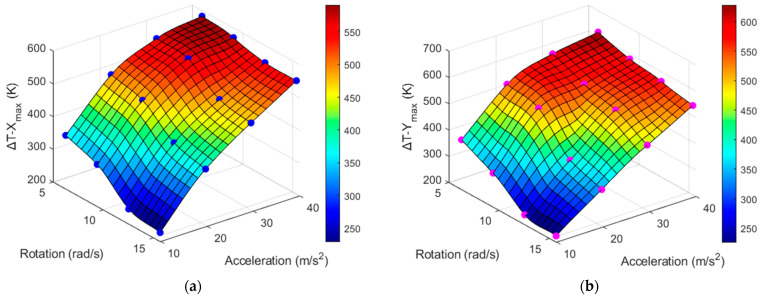
Data obtained after interpolation of Δ*T_X_max_* (**a**) and Δ*T_Y_max_* (**b**) w.r.t acceleration and rotation.

Because the temperature values obtained above were detected with the given acceleration and rotation values and changed linearly, cubic interpolation using MATLAB was employed to obtain more data. This is shown in Figure 17, where the data points are represented by mesh points.

**Figure 18:**
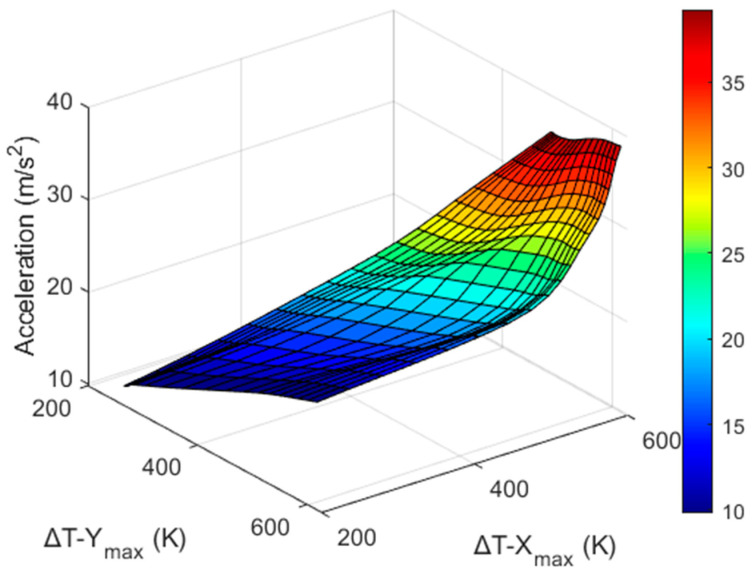
Graph to obtain *a* from Δ*T_y_* and Δ*T_x_* data measured around heater 3 (isometric drawing).

**Figure 19:**
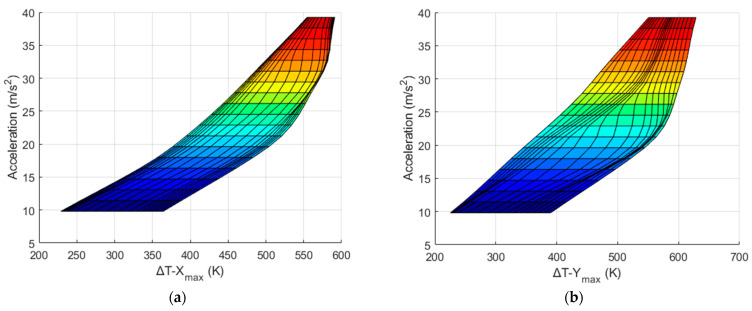
*a* w.r.t Δ*T_x_* (**a**) and Δ*T_y_* (**b**).

**Figure 20:**
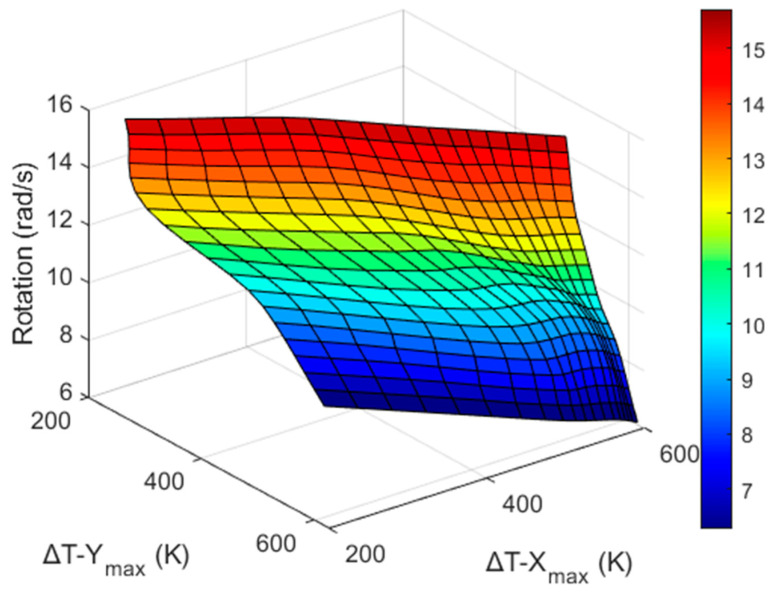
Graph to obtain ω from Δ*T_y_* and Δ*T_x_* data measured around heater 3 (isometric drawing).

**Figure 21:**
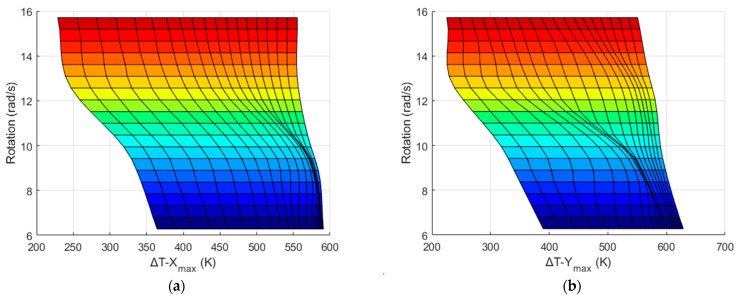
ω w.r.t Δ*T_x_* (**a**) and Δ*T_y_* (**b**).

Because the values Δ*T_X_max_* and Δ*T_Y_max_* can be measured by the X and Y sensors of a real thermal motion sensor, respectively, it is necessary to obtain an inverse function such that a real thermal motion sensor can calculate *a* and ω corresponding to the measured maximum temperature difference (Δ*T_max_*) values. This inverse function is represented by the equation below, and the inverse graphs are shown in Figure 18, Figure 19, Figure 20 and Figure 21.

(8) $$\left(a,\;\omega )\;=\;f^{-1}(\Delta T_{x},\;\Delta T_{y}\right)$$

For example, using the data shown in Figure 18 and Figure 19, a real thermal motion sensor can calculate the acceleration values from the values of Δ*T_y_* and Δ*T_x_* measured by this sensor. In summary, theoretically, from the results shown above, both rotation and acceleration can be determined simultaneously when a real thermal motion sensor rotates at any speed from 6.28 rad/s to 15.71 rad/s and accelerates in the *x* direction at any value from 1*g* to 4*g*. This range of rotational speed and acceleration can be further increased when more simulations are conducted. In this study, the data were collected only for the X and Y sensors around heater 3. Considering the other heater positions with their four surrounding sensors, similar results should be generated.

## 4. Discussion

In this study, we present a novel technique that can be implemented to measure multiple physical quantities simultaneously using a thermal motion sensor. We successfully implemented and demonstrated the simultaneous determination of acceleration and rotation.

The general method is described as follows. The idea is to define a relationship between multiple physical quantities that we are interested in and multiple outputs by computational simulations, as generalized below:

(output 1, output 2, …) = f (physical quantity 1, physical quantity 2, …)(9)

for each value of a physical quantity, there is a corresponding set of outputs (ΔT_max_ values in this case). However, the number of outputs should be the same as the number of physical input quantities. Therefore, the cross-axis sensitivity is useful. Once this relation has been obtained, the next step is to determine the inverse function of this relationship as:

(physical quantity 1, physical quantity 2, …) = f^−1^ (output 1, output 2, …) (10)

Using this relation, we can easily obtain a graphical inverse function for a specific range of physical quantities. Within this range, any value can be extracted based on the output. In this study, the maximum temperature values Δ*T_max_* around the *X* and *Y* sensors were obtained as two outputs corresponding to acceleration in the *x*-direction and rotation around the *z*-axis. The data of the inverse function can then be installed in the computing unit of a real thermal motion sensor so that the sensor can calculate the acceleration and rotation speed from the measured maximum temperature values Δ*T_max_* around the *X* and *Y* sensors.

Increasing the aerial system size with similar heater power of the thermal motion sensor should generate similar graph trends and performance parameters like sensitivity. As described in Section 1, sensitivity is related to the Grashof number, and in fluid mechanics, such groups are considered dimensionless groups such as Reynold’s number and Prandtl number. Therefore, temperature values and hence the inverse function need not be modified even if the size of the aerial system is increased or decreased. It is dependent on the applied physical quantities. The range of acceleration and rotation considered in the paper, however, is limited and needs to be further extended. If the range of the physical quantities is further increased, the modified inverse function can be added and programmed into the computing unit of the thermal motion sensor.

While using the concept of cross-axis sensitivity for a 3-axis thermal accelerometer, it has been mentioned by Nguyen [12] and Ogami [1] that “the same response can be observed for two accelerations with different magnitudes and opposite signs”, which means that two combinations of temperature outputs can determine a single acceleration. In our study, we observed acceleration in the *x*-direction and rotation around the *z*-direction, but even by considering X- and Y-sensor responses, because of cross-axis sensitivity, results were observed to have a unique combination of temperature outputs corresponding to accelerations of 1*g*–4*g* and rotational speeds of 6.28–15.71 rad/s. We define this characteristic of the results as the *uniqueness* of the solution. For distances of 0.1254 cm and 0.1433 cm between the heater and sensors, we observed areas of multiple solutions for changing rotational speeds. Therefore, for a defined range of input physical quantities, it is necessary to verify the results by simulations and find parameters that generate unique solutions.

In Section 3, we observed that a shorter distance between the heater and sensors results in better sensitivity and resolution. Six different sensor positions were considered, among which the shortest distance, 0.0179 cm, gave the most favorable results, while the largest one, 0.1433 cm, gave the least favorable results. This means that reducing this distance to the least possible value would generate better device performance. However, this distance is measured from the center of the heater to the center of the sensors; therefore, considering the practical aspects, there is a limit to how much this can be decreased. Therefore, a compromise is needed to choose the ideal distance between the heater and sensors.

In addition to sensitivity and resolution, another important factor that determines the quality of results is the frequency response. This is a measure of how quickly a device can respond to changes in acceleration and rotation speeds. To obtain a fast and wide frequency response, it is necessary to change the thermal physical properties of the gas medium. A large thermal conductivity (α) and small density (ρ) will accelerate thermal diffusion, which consequently facilitates heat balance in the cavity and provides a fast frequency response to the device. Multiple studies have been conducted on the frequency response of thermal accelerometers. Garraud [13] investigated the frequency response using analytical and CFD models. This could also be accomplished for a thermal motion sensor that measures multiple physical quantities, which will be our future work.

For our thermal motion sensor material, we used PVDF, which is known for its piezoelectric behavior. This implies that it can generate an electric charge in response to applied mechanical stress. Using this to our advantage, we may also define a new variable concerning pressure changes around the body. Using this application of the material, we can simultaneously measure an additional physical quantity, such as applied mechanical stress. However, PVDF has a high coefficient of thermal expansion (α), which limits its usage at higher temperatures. The properties and responsiveness of PVDF with respect to incorporation in a thermal motion sensor for pressure measurement will be investigated in our future work.

## 5. Conclusions

In this study, we presented a new method for simultaneously measuring multiple physical quantities using computational fluid dynamics. For any acceleration between 9.81 m/s^2^ and 39.24 m/s^2^ (1*g*–4*g*) and rotational speed of 6.28 rad/s–15.71 rad/s, this device can measure both quantities at the same time. This range can be expanded by additional computational simulations. The inverse function can be installed in the computing unit of a real thermal motion sensor so that the sensor can calculate the acceleration and rotation speed from the measured temperature values.

We also studied the effects of various parameters on the performance of the device, and it was observed that by reducing the distance between the heater and sensor positions, the sensitivity and resolution may be improved. In addition, using a gas medium with a high density and low viscosity may improve the sensitivity and resolution of the device.

In the future, using the same technique, other physical quantities, such as vibrations, can also be analyzed. Here, we studied a thermal motion sensor, and by modifying it and using cross-axis sensitivity, we were able to measure multiple physical quantities simultaneously. However, we believe that the idea presented here can also be applied to other sensors, such as stress sensors or vibration sensors. In addition to the performance parameters mentioned in this study, i.e., sensitivity, resolution, and *uniqueness* of the solution, a frequency response analysis should also be performed to verify the quality of the results. The computational model stated here should also be validated experimentally.

## Figures and Tables

**Table 1:** Gas medium properties at 50 °C (adapted from [8]).

	Density (kg/m^3^)	Specific Heat (J/kg·K)	Kinematic Viscosity (×10^−6^) (m^2^ /s)	Thermal Diffusivity (×10^−4^) (m^2^ /s)	Thermal Conductivity (W/m·K)
Air	1.092	1007	19.6	0.248	0.02735
N_2_	1.0564	1042	17.74	0.249	0.02746
CO_2_	1.6597	866.6	9.71	0.129	0.01858

**Table 2:** Calculated Gr and Pr numbers.

	Air	N_2_	CO_2_
Gr	7.44 × 10^−3^	8.07 × 10^−3^	4.24 × 10^−2^
Pr	7.16 × 10^−4^	6.46 × 10^−4^	5.22 × 10^−4^

**Table 3:** Mechanical properties of MEMS materials.

Material	Thermal Conductivity (W/(m·K))	Specific Heat Capacity (J/(kg·K))	Density (kg/m^3^)
PVDF	0.2	1500	1780
Polyimide	0.1	1100	1420

**Table 4:** Δ*T_X_max_* values around heater 3.

**Δ*T*_*X_max_* (K)**		**2π**	**3π**	**4π**	**5π**
	**1*g***	364.3	329.2	247.0	228.9
	**2*g***	503.9	479.5	403.5	377.0
	**3*g***	568.4	561.1	489.9	472.2
	**4*g***	591.0	579.4	556.7	555.4

**Table 5:** Δ*T_Y_max_* values around heater 3.

**Δ*T*_*Y_max_* (K)**		**2π**	**3π**	**4π**	**5π**
	**1*g***	389.7	330.2	238.6	225.4
	**2*g***	544.4	520.5	389.8	345.0
	**3*g***	602.8	554.3	523.5	457.1
	**4*g***	628.6	593.4	575.6	550.7
